# Functional consequences of non-equilibrium dynamics caused by antisymmetric and symmetric learning rules

**DOI:** 10.1186/1471-2202-16-S1-P96

**Published:** 2015-12-18

**Authors:** Dmytro Grytskyy, Markus Diesmann, Moritz Helias

**Affiliations:** 1 Institute of Neuroscience and Medicine (INM-6) and Institute for Advanced Simulation (IAS-6), Jülich Research Centre and JARA, Jülich, Germany; 2 Department of Psychiatry, Psychotherapy and Psychosomatics, Medical Faculty, RWTH Aachen University, Aachen, Germany; 3 Department of Physics, Faculty 1, RWTH Aachen University, Aachen, Germany

## 

Connectivity in the brain is asymmetric, which is evident by Dale's principle of excitatory and inhibitory neurons. As a consequence, biologically realistic neuronal networks cannot be in thermodynamic equilibrium [1]. Even in a stationary state, probability fluxes perpetuate, leading to non-equilibrium steady states [2,3]. We here investigate the computational consequences of non-equilibrium dynamics for synaptic plasticity and learning. Formulating the stochastic dynamics in sparsely connected networks of non-linear Langevin equations in terms of path integrals [4], we show that biologically plausible correlation-sensitive plasticity rules follow from first principles. We investigate two different rules: 1.) Maximizing a measure of irreversibility [2] by gradient descent with respect to the weights, we obtain a local learning rule sensitive to the derivative of the covariance between the pre- and postsynaptic neuron. The obtained rule can be interpreted as spike-timing dependent plasticity (STDP) [5,6] with a narrow antisymmetric learning window. We show that the learning rule increases synaptic weights in the direction of the (direct or indirect) causal influence between a pair of neurons. In this way indirect causal relationships are transformed into strengthened direct connections. An instability of the state with vanishing weights manifests itself as spontaneous symmetry breaking and the divergence of connection weights. 2.) We investigate a rule that is sensitive to the zero-time-lag covariance. It can be considered as a complement to the first rule approximating STDP with a symmetric narrow learning window. This rule results in the strengthening of loops, of mutual connections between neurons getting common input, and of indirect (also backward) connections between neurons a and b if a influences b. We derive analytical expressions that describe these effects quantitatively. We show how nonlinearities in the neuronal transmission naturally stabilize synaptic weights and how the limited dynamic range of neuronal activity mediates competition between synapses. The work elucidates the tight connection between measures of reversible and irreversible dynamics and the structures resulting from learning rules that optimize either of the two.

## References

[B1] Sompolinsky H Statistical mechanics of neuronal networks Physics Today 1988 70 80

[B2] Seifert U Stochastic thermodynamics, fluctuation theorems, and molecular machines Rep Prog Phys 2012 75 126001 doi:10.1088/0034-4885/75/12/126001 23168354 10.1088/0034-4885/75/12/126001

[B3] Evans DJ Morriss GP Statistical Mechanics of Non Equilibrium Liquids 1990 London: Academic Press

[B4] Wio HS Colet P San Miguel M Path-integral formulation for stochastic processes driven by colored noise Phys Rev A 1989 40 7312 7324 9902148 10.1103/physreva.40.7312

[B5] Bi GQ Poo MM Synaptic modifications in cultured Hippocampal neurons: dependence on spike timing, synaptic strength, and postsynaptic cell type J Neurosci 1998 18 10464 72 9852584 10.1523/JNEUROSCI.18-24-10464.1998 PMC6793365

[B6] Morrison A Diesmann M Gerstner W Phenomenological models of synaptic plasticity based on spike timing Biol Cybern 2008 98 459 478 18491160 10.1007/s00422-008-0233-1 PMC2799003

